# BMPR-1B, BMP-15 and GDF-9 genes structure and their relationship with litter size in six sheep breeds reared in Egypt

**DOI:** 10.1186/s13104-020-05047-9

**Published:** 2020-04-10

**Authors:** Ahmed. A. Saleh, M. H. Hammoud, Nasraa A. Dabour, E. E. Hafez, Mahmoud A. Sharaby

**Affiliations:** 1 College of Animal Science and Technology, Southwest University, Chongqing Key Laboratory of Forage & Herbivore, Chongqing Engineering Research Center for Herbivores Resource Protection and Utilization, Beibei, 400716 Chongqing People’s Republic of China; 2 Animal and Fish Production Department, Faculty of Agriculture (Alshatby), Alexandria University, Alexandria, Egypt; 3 Dairy Science and Technology Department, Faculty of Agriculture (Alshatby), Alexandria University, Alexandria, Egypt; 4 Plant Protection and Bio-molecular diagnosis Department, Arid Lands Cultivation Research Institute (ALCRI), The City of Scientific Research and Technology Applications, Alexandria, Egypt

**Keywords:** Fec-B, BMP-15, GDF9, RFLP, SSCP, DNA sequencing

## Abstract

**Objective:**

The aim of this work was to investigate three different mutations; Fec-B, FecX^G^, Fec-G^H^ at three candidate genes; Bone morphogenetic protein receptor IB, Bone morphogenetic protein 15 and Growth Differentiation Factor 9, respectively, in six sheep breeds reared in Egypt namely; Rahmani, Barki, Rahmani X Barki cross, Awassi, Awassi X Suffolk cross, and Ossimi and their association with litter size.

**Results:**

Genomic DNA of 132 sheep was investigated for the Fec-B, FecX^G^, and Fec-G^H^ mutations by Restriction Fragment Length Polymorphism, Single-Stranded Conformation Polymorphism and DNA sequencing. The results revealed that all breeds did not carry Fec-B mutation. On the other side, the mutations of FecX^G^, and Fec-G^H^ were detected in Rahmani, and Rahmani X Barki cross which is associated with the high twinning rate/litter size of Rahmani (1.28) and Rahmani X Barki cross (1.22). While, the average litter size for other breeds had almost a constant values rate over six parities, ranging between 1.00 and 1.04.

## Introduction

The productive and reproductive traits are influenced by a large number of minor genes scattered across the genome [1]. Selection for these genes requires special tools, pedigree assisted selection and progeny testing have been used widely to improve many of these traits [2]. Fertility, though being one of these traits, it possesses lowly heritable nature due to strong environmental influences. Additionally, fertility is controlled by the Bone morphogenetic protein receptor IB (BMPR-IB), bone morphogenetic protein 15 (BMP15) and Growth Differentiation Factor 9 (GDF9) genes. These major genes were found to participate in determining sheep fertility behaviour [2–8]. Presence of mutations in these genes increase ovulation and lambing rates dramatically with ewes producing 2, 3, or even more lambs per parturition [4], enhance reproductive endocrinology [9], and ovarian activity and product of litter size (LS), body mass and other organs development [10–13]. Several previous studies discussed the relationship of Fec-B, Fec-G^H^, and FecX^G^ mutations in the above-mentioned genes with major effects on LS and ovulation rate in sheep [2, 6, 7, 14, 15].

The objectives of this work were to examine the presence of BMPR-1B (Fec-B), BMP-15 (Fec-G^H^) and GDF9 (FecX^G^) mutations and their association with LS in six sheep breeds reared in Egypt using forced PCR-Restriction Fragment Length Polymorphism (PCR–RFLP), Single-Strand Conformational Polymorphism (SSCP) and DNA sequencing.

## Main text

### Materials and methods

#### Animals

Samples from six sheep breeds viz; Rahmani (n = 20 ewes), Barki (n = 20 ewes), Awassi (n = 10 rams and ewes), Awassi X Suffolk (n = 8 rams and ewes), Ossimi (n = 5 rams and ewes) and Rahmani X Barki cross (n = 69 rams and ewes) were obtained from three different geographical regions at the northern region of Egypt, namely; Alexandria University ‘experimental station’ (GPS: 31.206208, 29.919704)—Matrouh governorate (GPS: 31.336924, 27.205762) and Sakha (GPS: 31.087032, 30.948859). The data records for LS have been investigated for each breed for six parities.

#### DNA isolation and amplification

Total genomic DNA was extracted from sheep blood samples with (QIAGEN kit, Germany). The DNAs were separated by electrophoresis on 0.8–1% agarose in 0.5 × TBE buffer [16] which contained around 0.5 μg/ml ethidium bromide. The electrophoresis run was performed utilizing apparatus with power supply and visualized by ultra-violet transilluminator and Gel documentation system (Alpha-chem. Imager, USA). The specificities of the PCR primers targeting BMPR-1B, BMP-15 and GDF-9 genes were previously tested by Wilson et al. [17], and Hanrahan et al. [18] (Additional file 2: Table S1). The amplifications were performed using (iQ SYBR G-S.mix, USA), 10 p.mol of each primer and 80 ng of genomic DNA processed under the amplification conditions shown in (Additional file 2: Table S2). The amplification was carried out using a Thermo-cycler Gene Amp 6700 (Bio-system, USA).

#### Restriction fragment length polymorphism (RFLP)

The RFLP technique was used to detect the differences between sheep breeds, utilizing the PCR products of target genes. The PCR products of BMPR-1B gene were digested with *AvaII* (Jena Bioscience, Germany) and of GDF-9 with *ASP*-*I* and *Hinf*-*I* (Bio-search Technologies, USA) separately. The reaction volume was 25 μl, consisting of 10 μl PCR product, 2 μl 10× digestion buffer, 12 μl H_2_O, 5 units restriction enzyme (5 unit/1 μl). All reactions were incubated at 37 °C for 14–16 h. 20 μl of each reaction were separated by electrophoresis on 3–4% agarose gel. Similarities and differences between breeds were detected by inspecting RFLP patterns.

#### Single-strand conformational polymorphism (SSCP)

The SSCP analysis was employed according to Bastos et al. [19] to detect the possible mutations in BMP-15 gene. Aliquots of 7–10 μl PCR products were mixed with 2–3 μl of SSCP dye (95% formamide, 0.025% bromophenol blue, 0.05% xylene-cyanole and 0.5 M pH 8.0 EDTA, Mixture by vortexing and then storing at − 20 °C), incubated at 95 °C for 5 min and then chilled on ice. Denatured DNA was loaded on 10% PAGE gel (90 mm × 75 mm × 0.75 mm). Electrophoresis was performed on 0.5× TBE buffer at room temperature using the constant voltage of 70 to 90 v for 5–6 h.

#### Nucleotide sequence analysis

The sequence analysis was carried out for GDF-9 gene by DNA Sequencer (ABI Prism 3100 apparatus, USA). Database similarity searches were performed with (NCBI) (http://www.ncbi.nlm.nih.gov). The resulted sequences were analyzed using Blast 2.0, MEGA 5.05, to detect Single-Nucleotide Polymorphism (SNPs) among different samples. The sequences of GDF-9 gene for studied animals were deposited in GenBank (Accession Numbers: KT357481.1, KT357482.1, KT357484.1, KT357483.1, KT357485.1, and KT357486.1) for Rahmani, Barki, Rahmani X Barki cross, Awassi, Awassi X Suffolk cross, and Ossimi, respectively. Analysis of translated amino acid of GDF-9 gene sequences of tested sheep breeds were generated using ExPASy program.

#### Statistical analysis

Data records of LS were tested for normality with the Shapiro–Wilk test from the UNIVARIATE procedure (SAS, 2004), and results indicated that all data were distributed normally [Shapiro–Wilk test (W) ≥ 0.90]. Also, the GLM procedure of SAS was used to determine the effects of breed on ewes` prolificacy (or LS of ewes) according to the following model:

$${\text{Y}}_{\text{ij}} = {\upmu }+ \;{\text{B}}_{\text{i}} \; + \;{\text{e}}_{\text{ij}}$$

Where: Y_ij_ = the ewes` prolificacy, µ = the overall mean, B_i_ = the fixed effect of ith breed, and e_ij_ = the residual error. Significant differences among means within each LS were tested using least significant difference (LSD_0.05_).

### Results and discussion

#### Amplification, manipulation, and digestion

This research note concerns mainly the differentiation between six sheep breeds for Fec-B, Fec-G^H^ and FecX^G^ mutations. It also spotlights the association between polymorphism of these mutations and their relationship with LS. PCR amplification for the tested sheep breeds produced an amplified 190 bp fragment for BMPR-1B (Additional file 1: Figure S1a), 141 bp fragment for BMP-15 (Additional file 1: Figure S1b), and 462 bp fragment for GDF-9 (Additional file 1: Figure S1c).

#### BMPR-1B gene

The PCR products of BMPR-1B gene obtained for tested animals after digestion with *AvaII* (Fig.S2a) revealed only one pattern for one DNA fragment sized 190 bp and indicated the absence of a restriction site of *AvaII*. These results were in agreement with those found by Abulyazid et al. [20] on a total of 22 crossbred Egyptian sheep (11 twins producing a female, 7 single lambs producing a female, and 4 male). Also, was in agreement with the results of EL–Hanafy and El-Saadani [21]. who concluded an absence of the gene in five Egyptian sheep breeds. On the other hand, Guan et al. [22] found that; 7 out of 9 sheep breeds, namely, Suffolk, Charolais, Hu, Chinese Merino prolific meat strain, Dorset, Romney Hills, Merino and crossbreed of Suffolk × Chinese Merino as well as Dorset × Chinese Merino were of wild-type (++) with 190 bp for the restriction pattern of Fec-B gene. They observed a positive relationship between Fec-B mutation and LS in Chinese Merino prolific meat strain. The BB homozygous mutant genotype sized around 2.84 ± 0.74 LS (P > 0.05) which was greater (P < 0.01) than that of ewes with ++ genotype (1.23 ± 0.68) LS. Also, the ewes with BB genotype produced 0.5 LS lambs more than those with B + genotype, though this difference was not significant.

#### GDF-9 gene

Digestion of PCR fragments of GDF-9 gene which was performed using two different restriction enzymes, *ASP*-*I* and *Hinf*-*I* separately produced different PCR profiles. The *ASP*-*I* profile produced two fragments of 280 and 182 bp for all tested samples (Additional file 1: Fig. S2b_1_) and the *Hinf*-*I* pattern gene produced 300, 110 and 52 bp bands for all tested breeds and crosses, (Additional file 1: Fig. S2b_2_). The two digestion profiles indicated the presence of one genotype for all tested breeds. Therefore, to identify the genotypic diversity of GDF-9 (Fec-G^H^) gene among the studied animals the complete sequences of all amplified fragments of this gene were performed for mutation detection.

#### Nucleotide sequence and amino acid sequence comparisons for GDF-9 gene

**Fig. 1:**
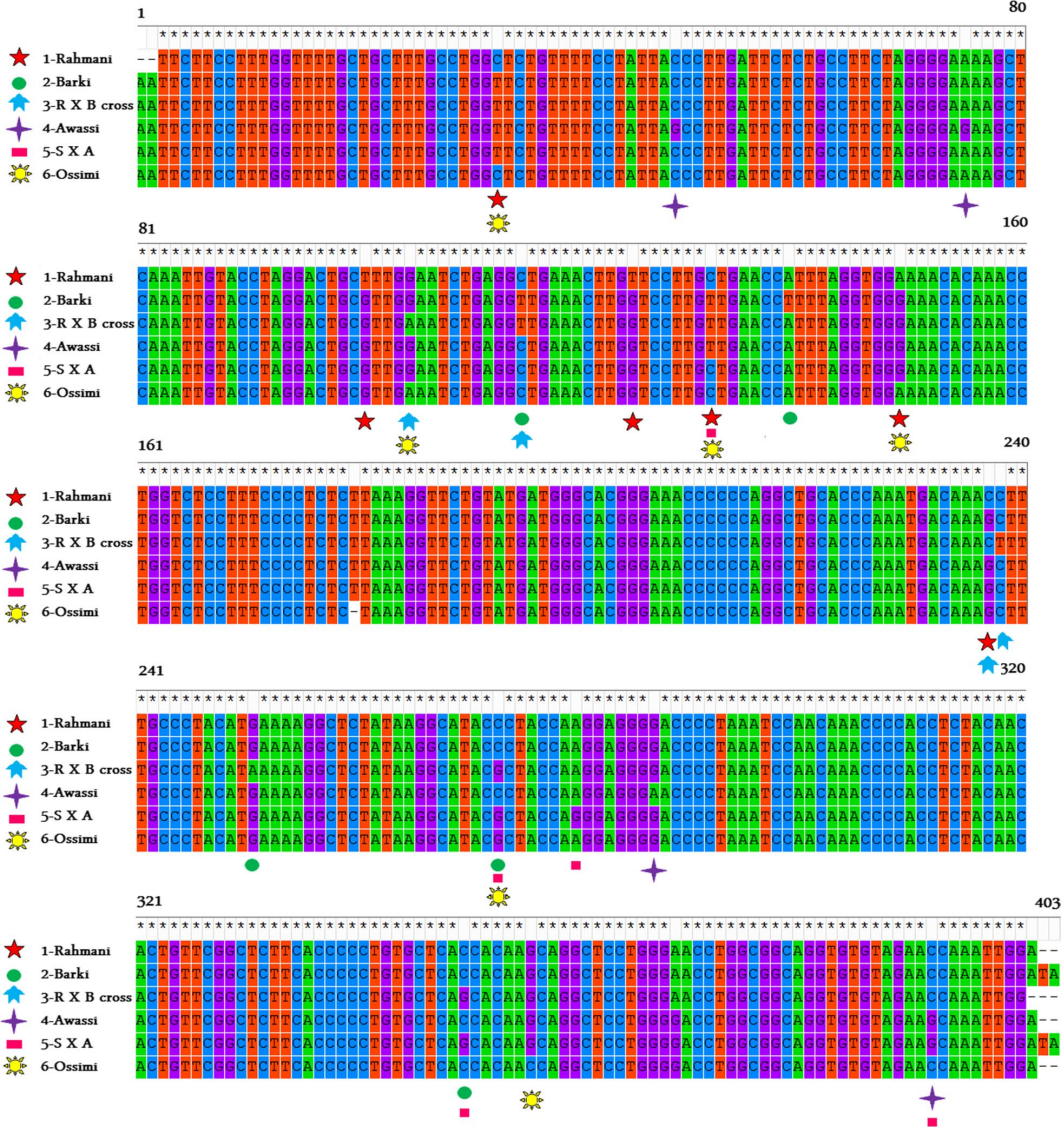
Nucleotide sequence comparison of amplified GDF-9 gene of tested sheep using MEGA 5.05 (1993–2011) and its site https://mega.software.informer.com/5.0. *RXB* Rahmani X Barki cross and *SXA* Awassi X Suffolk cross

Nucleotide sequencing of the amplified fragment of GDF-9 gene of Rahmani, Barki, Rahmani X Barki cross, Awassi, Awassi X Suffolk cross, and Ossimi, respectively were performed and submitted to the NCBI GenBank (Accession Numbers: KT357481.1, KT357482.1, KT357484.1, KT357483.1, KT357485.1, and KT357486.1) (Additional file 1: Fig. S3). The nucleotide sequence analysis indicated that the percent distances of GDF-9 gene fragment of sheep breeds shown in (Additional file 2: Table S3), (Fig. 1) were generated by using MEGA 5.05.

**Fig. 2:**
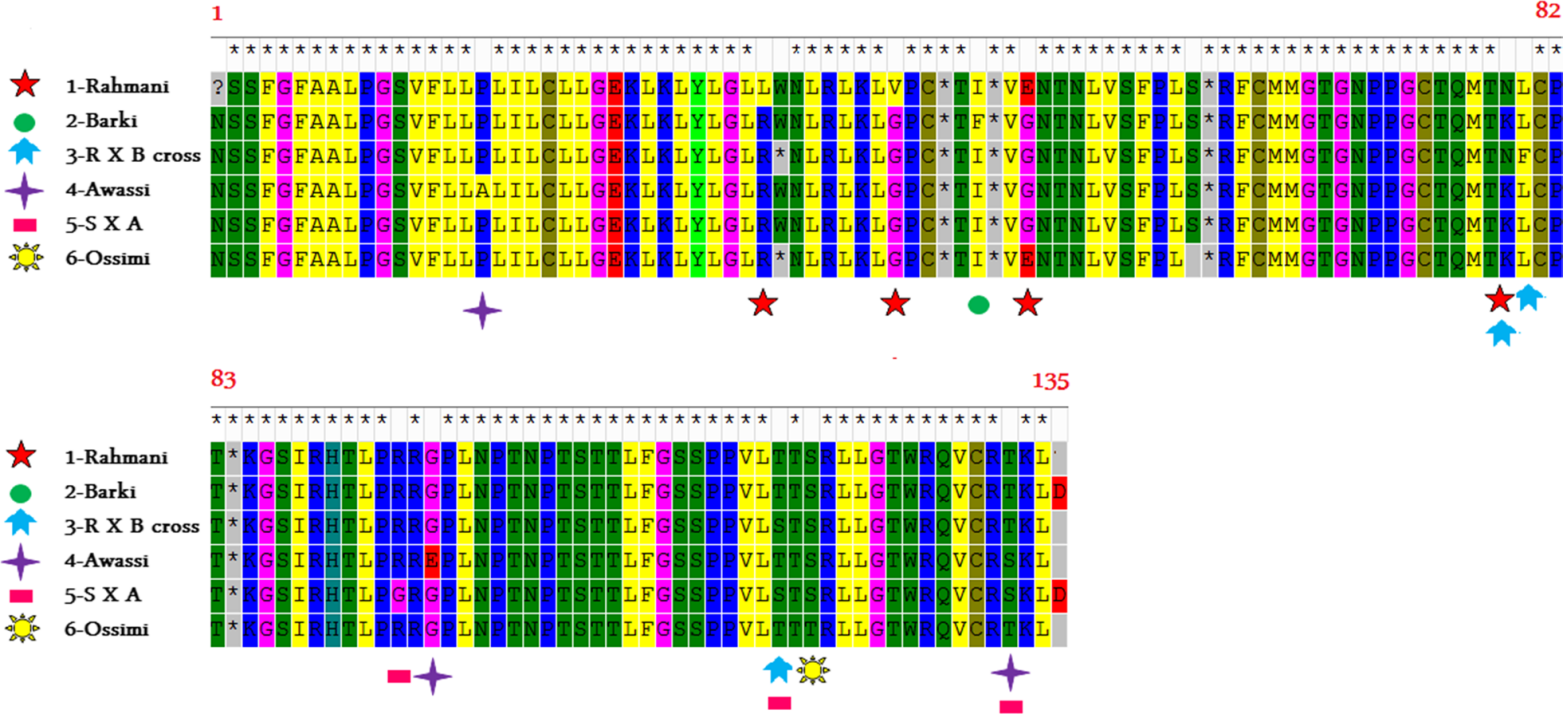
Comparative analysis of amino acid sequence of GDF-9 gene of tested sheep breeds using ExPASy program and its site (http://web.expasy.org/translate) and MEGA 5.05 (1993–2011) (https://mega.software.informer.com/5.0), *RXB* Rahmani X Barki cross and *SXA* Awassi X Suffolk cross

The sequence of the amplified GDF-9 gene fragments of tested sheep breeds as generated by ExPASy program and the comparison of amino acids obtained by MEGA 5.05 are in (Fig. 2). The amino acid sequence of Rahmani breed was different from that of other breeds by four amino acids. The different amino acids were (Lysine, Valine, Glutamic and Asparagin) in Rahmani instead of (Arginine, Glycine, Glycine, and Lysine) in other breeds, while in Rahmani X Barki cross the amino acid sequence was different from that of other breeds by three amino acids, the different amino acids were (Asparagine, Phenylalanine, and Serine), instead of (Lysine, Leucine, and Threonine). Otherwise, Barki breed amino acid sequence was different from that of other breeds in one amino acid, only that was (Phenylalanine) instead of (Isoleucine). The changing amino for all breeds is shown in (Additional file 2: Table S4). Hence, there was approximately (97.01%) similarity in the amino acid sequence between tested animals.

On another level, Noshahr and Rafat [23]. studied the RFLP performed to detect the genetic diversity of GDF-9 (Fec-G^H^) in Moghani sheep population and identified three different patterns. The first pattern yielded three DNA fragment-sized 52, 156 and 254 bp and was classified as (FecG +). The second pattern yielded two DNA fragments of 52 and 410 bp and was classified as (FecG-). While the third pattern produced four DNA fragments of 52, 156, 254 and 410 bp, the carriers of this pattern were classified as heterozygous for the mutation GDF-9 (Fec-G^H^) gene.

Therefore, the GDF9 gene can be considered as a possible candidate for increased LS in sheep [24]. In the present study, genetic variation were found within the GDF9 gene based on nucleotide sequence for Rahmani and its cross with Barki.

### BMP-15 gene

#### Genotyping of BMP-15 gene by SSCP technique

Images of PCR products of SSCP technique performed to identify polymorphism and detect the alleles within BMP-15 gene, genotypes are in (Additional file 2: Table S5). The SSCP results for BMP-15 PCR product of separate breeds showed homozygous genotype (GG) of BMP-15 gene for Rahmani while, Rahmani X Barki cross had the three different genotypes (GG, G + , and ++), on the other side, there is only homozygous genotype (++) for Barki, Awassi, Awassi X Suffolk cross, and Ossimi. Similar heterozygous genotype for BMP-15 was detected in small tailed Han sheep when two genotypes, ++ (111 bp/111 bp) and G + (141 bp/111 bp) were identified by SSCP [6, 7]. Detection of mutations for BMP-15 gene of Rahmani and its cross with Barki may form an advantage for the Egyptian sheep industry. Rahmani sheep are characterized by high prolificacy that could be associated with G allele of BMP-15 gene detected in that particular breed and its cross with Barki. Such high prolificacy could be due to the regulatory mechanism of the G allele of BMP-15 gene [5].

#### The relationship between fertility and the prevalence of BMP-15 (Fec-X^G^) and GDF-9 (Fec-G^H^) genotypes

Ovine BMP-15 gene plays a vital role in the growth and differentiation of early ovulation follicles and increased ovulation rate and LS under heterozygous conditions [25]. Interestingly, homozygous carriers for this mutation were infertile in some sheep breeds [7]. According to the results of nucleotide sequencing for GDF-9 gene and SSCP technique for BMP-15 gene, Rahmani breed and its cross with Barki carried the mutations Fec-G^H^ of GDF-9 and Fec-X^G^ of BMP-15 genes. Additionally, the replacement of amino acids for GDF9 may enhance the high rate of twinning [18, 26]. This theory agrees with our results on the twinning rate in Rahmani and its cross with Barki compared to other breeds. High rate of twinning of 1.28 and 1.22 was recorded for Rahmani and its cross with Barki breeds, respectively. While the average of twinning rate for Barki, Ossimi, Awassi, and its cross with Suffolk had almost a constant values rate over six parities, ranging between 1.00 and 1.04 (Table 1).

**Table 1:** Prolificacy means for some sheep breeds in different parities

Breeds	Parity						Overall
	1	2	3	4	5	6	
Rahmani	1.17	1.00	1.33	1.50	1.17	1.50	1.28^b^
Barki	1.00	1.00	1.00	1.00	1.00	1.17	1.03^a^
RXB	1.00	1.22	1.47	1.13	1.33	1.17	1.22^b^
Awassi	1.00	1.00	1.00	1.00	1.00	1.00	1.00^a^
SXA	1.00	1.00	1.00	1.00	1.00	1.00	1.00^a^
Ossimi	1.00	1.00	1.00	1.00	1.20	1.00	1.04^a^

^a, b^Means with different superscript letters in the same column are significantly different

*RXB* Rahmani X Barki cross, *SXA* Awassi X Suffolk cross

### Limitations

The prolificacy genotypes at genes BMPR-1B, GDF-9 and BMP-15 were found so far in the genomes of many prolific breeds throughout the world. More research on these genes in Egyptian sheep breeds is required for detection of more polymorphisms, comparisons of gene sequencing and tracing the evolutionary relatedness for gene sights among sheep breeds. More investigations to confirm and apply those advantages are necessary. Also, detection of useful genes or mutations as a primary step before transferring them to another breed, composite, or line either by crossing or even by more sophisticated techniques should help achieving the betters of their presence and avoid the damage that they may cause if compiled wrongly.

## Supplementary information

**Additional file 1: Fig.S1** (**a**): PCR amplification of BMPR-1B gene (190 bp), (**b**): BMP-15 gene (141 bp), and (**c**): GDF-9 gene (462 bp), for; Rahmani (R), Barki (B), Awassi (A), Rahmani X Barki cross (C), Awassi X Suffolk cross (S), and Ossimi (O). M, 50 bp/ 100 bp DNA ladder. **Fig.S2** (**a**): The PCR products of the BMPR-1B (Fec-B) gene from the genomic DNA of tested breeds and digested by *AvaII*. M; 50 bp DNA ladder, M*; 100 bp DNA ladder. (**b**): Digestion pattern of PCR amplification of the GDF-9 (Fec-GH) gene from the genomic DNA of tested breeds. (**b1**) and (**b2**) digestion profile with *ASP-I* and *Hinf-I* respectively, for; Rahmani (R), Barki (B), Awassi (A), Rahmani X Barki cross (C), Awassi X Suffolk cross (S), and Ossimi (O). M, 100 bp /50 bp DNA ladder, respectively. **Fig.S3** (A) - A 462 bp sequence of GDF-9 gene of Rahmani breed *(NCBI accession no. KT357481.1)*, (B) - A 462 bp sequence of GDF-9 gene of Barki breed *(NCBI accession no. KT357482.1)*, (C) - A 462 bp sequence of GDF-9 gene of Rahmani X Barki cross breed *(NCBI accession no. KT357484.1)*, (D) - A 462 bp sequence of GDF-9 gene of Awassi breed *(NCBI accession no. KT357483.1)*, (E) - A 462 bp sequence of GDF-9 gene of Awassi X Suffolk cross breed *(NCBI accession no. KT357485.1)*, (F) - A 462 bp sequence of GDF-9 gene of Ossimi breed *(NCBI accession no. KT357486.1)*.

**Additional file 2: Table S1** The listing primer and sequence (5’→3’) of BMPR-1B, BMP-15, and GDF-9 genes. **Table S2** Cycles conditions of PCR. **Table S3** Nucleotide sequence distances: percent similarity (above diagonal), percent distance (below diagonal); of GDF-9 gene of tested sheep breeds. **Table S4** The changed amino acids in tested sheep breeds. **Table S5** Genotyping and allele frequencies analysis of the studied sheep breeds for BMPR-1B, and BMP-15 genes.

## Data Availability

All data generated or analyzed during this study are included in this manuscript and its Additional files: (1, 2).

## Abbreviations

BMPR-IB: Bone morphogenetic protein receptor-IB
GDF-9: Growth differentiation factor-9
BMP-15: Bone morphogenetic protein-15
PCR: Polymerase chain reaction
RFLP: Restriction fragment length polymorphism
SSCP: Single-strand conformational polymorphism
