# Supplementary material for: BMPR-1B, BMP-15 and GDF-9 genes structure and their relationship with litter size in six sheep breeds reared in Egypt
Source: BMC Res Notes. 2020 Apr 10;13:215. doi: 10.1186/s13104-020-05047-9 (PMC7160958; doi:10.1186/s13104-020-05047-9)
Supplement: Supplementary file 1 — Additional file 1: Fig.S1 (a): PCR amplification of BMPR-1B gene (190 bp), (b): BMP-15 gene (141 bp), and (c): GDF-9 gene (462 bp), for; Rahmani (R), Barki (B), Awassi (A), Rahmani X Barki cross (C), Awassi X Suffolk cross (S), and Ossimi (O). M, 50 bp/ 100 bp DNA ladder. Fig.S2 (a): The PCR products of the BMPR-1B (Fec-B) gene from the genomic DNA of tested breeds and digested by AvaII. M; 50 bp DNA ladder, M*; 100 bp DNA ladder. (b): Digestion pattern of PCR amplification of the GDF-9 (Fec-GH) gene from the genomic DNA of tested breeds. (b1) and (b2) digestion profile with ASP-I and Hinf-I respectively, for; Rahmani (R), Barki (B), Awassi (A), Rahmani X Barki cross (C), Awassi X Suffolk cross (S), and Ossimi (O). M, 100 bp /50 bp DNA ladder, respectively. Fig.S3 (A) - A 462 bp sequence of GDF-9 gene of Rahmani breed (NCBI accession no. KT357481.1), (B) - A 462 bp sequence of GDF-9 gene of Barki breed (NCBI accession no. KT357482.1), (C) - A 462 bp sequence of GDF-9 gene of Rahmani X Barki cross breed (NCBI accession no. KT357484.1), (D) - A 462 bp sequence of GDF-9 gene of Awassi breed (NCBI accession no. KT357483.1), (E) - A 462 bp sequence of GDF-9 gene of Awassi X Suffolk cross breed (NCBI accession no. KT357485.1), (F) - A 462 bp sequence of GDF-9 gene of Ossimi breed (NCBI accession no. KT357486.1). [file 13104_2020_5047_MOESM1_ESM.pdf]

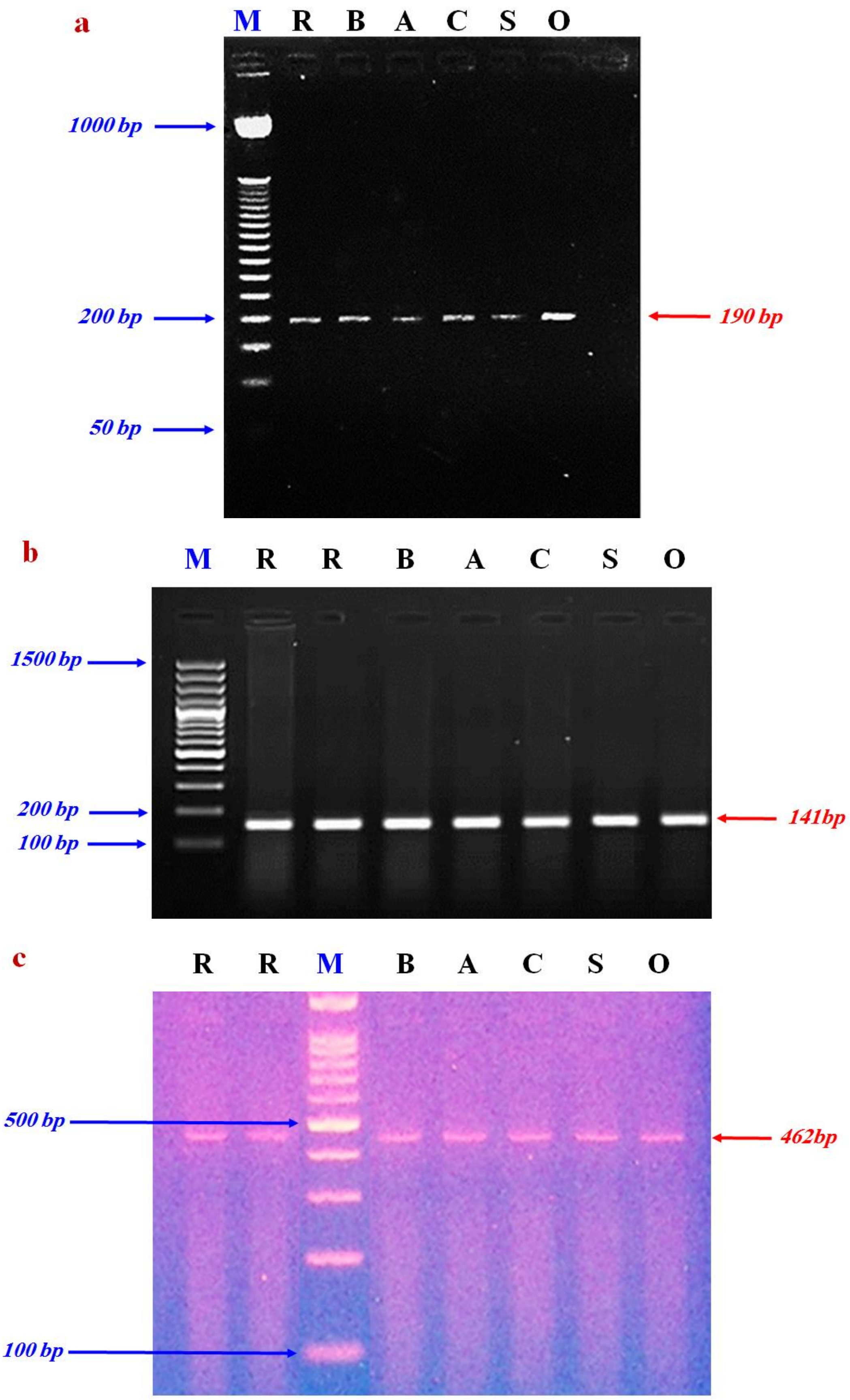

Fig.S1 (**a**)**:** PCR amplification of BMPR-1B gene (190 bp), (**b**)**:** BMP-15 gene (141 bp), and (**c**)**:** GDF-9 gene (462 bp), for; Rahmani (R), Barki (B), Awassi (A), Rahmani X Barki cross (C), Awassi X Suffolk cross (S), and Ossimi (O). M, 50 bp/ 100 bp DNA ladder.

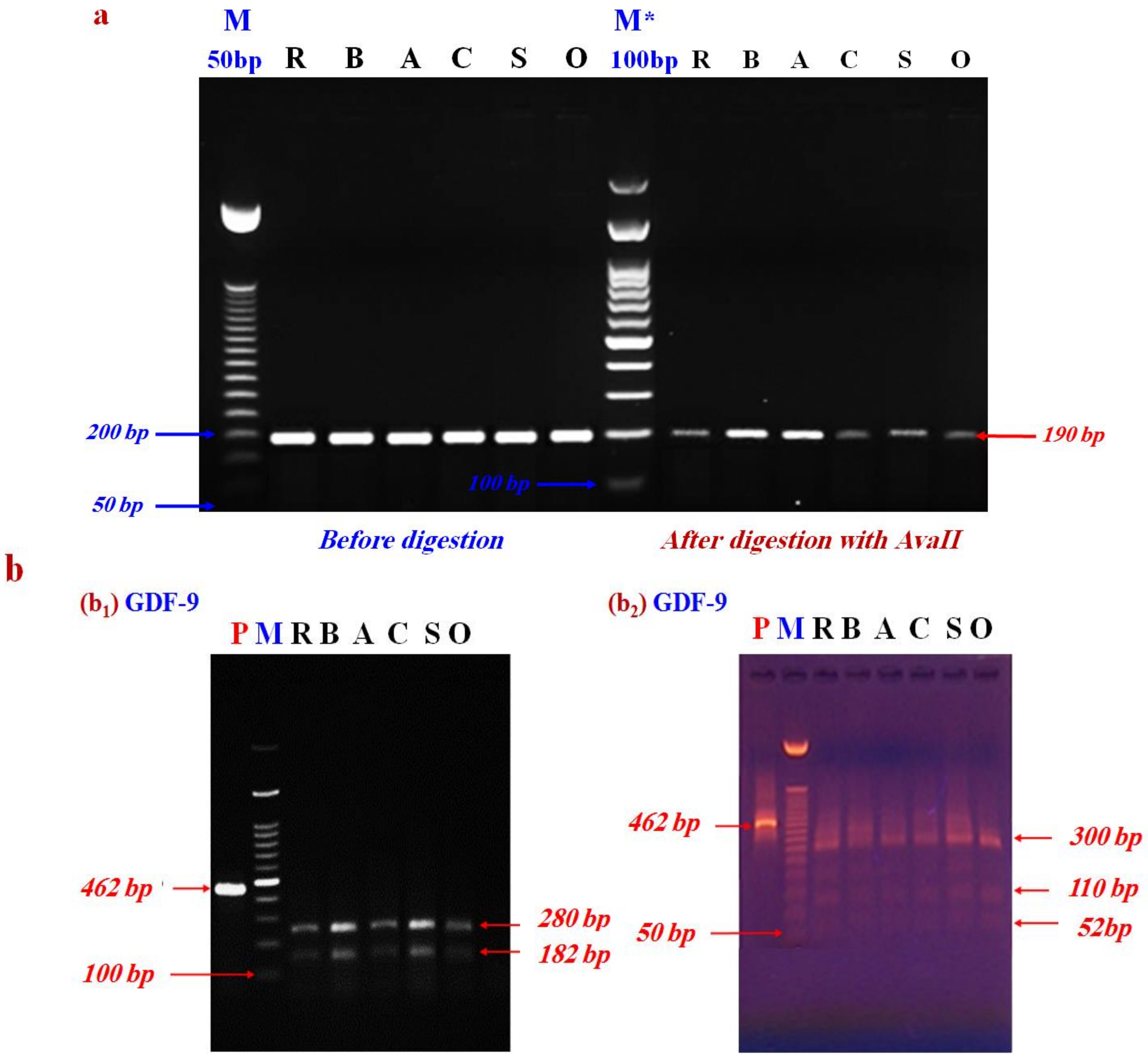

Fig.S2 (**a**)**:** The PCR products of the BMPR-1B (Fec-B) gene from the genomic DNA of tested breeds and digested by *AvaII*. M; 50 bp DNA ladder, M*; 100 bp DNA ladder. (**b**)**:** Digestion pattern of PCR amplification of the GDF-9 (Fec-G^{H}) gene from the genomic DNA of tested breeds. (**b_1**) and (**b_2**) digestion profile with *ASP-I* and *Hinf-I* respectively, for; Rahmani (R), Barki (B), Awassi (A), Rahmani X Barki cross (C), Awassi X Suffolk cross (S), and Ossimi (O). M, 100 bp /50 bp DNA ladder, respectively.

A

```
  1 ttcttccttt ggttttgctg ctttgcctgg ctctgttttc ctattaccct tgattctctg
 61 ccttctaggg gaaaagctca aattgtacct aggactgctt tggaatctga ggctgaaact
121 tgttccttgc tgaaccattt aggtggaaaa cacaaacctg gtctcctttc ccctctctta
181 aaggttctgt atgatgggca cgggaaaccc cccaggctgc acccaaatga caaanctttg
241 ccctacatga aaaggctcta taaggcatac cctaccaagg aggggacccc taaatccaac
301 aaaccccacc tctacaacac tgttcggctc ttcacccccct gtgctcacca caagcaggct
361 cctgggaacc tggcggcagg tgtgtagaac caaattgga
```

B

```
  1 aattcttcct ttggttttgc tgctttgcct ggttctgttt tcctattacc cttgattctc
 61 tgccttctag gggaaaagct caaattgtac ctaggactgc gttggaatct gaggttgaaa
121 cttggtcctt gttgaacctt ttaggtggga aacacaaacc tggtctcctt tcccctctct
181 taaaggttct gtatgatggg cacgggaaac cccccaggct gcacccaaat gacaaagctt
241 tgccctacat gaaaaggctc tataaggcat accctaccaa ggaggggacc cctaaatcca
301 acaaacccca cctctacaac actgttcggc tcttcacccc ctgtgctcac cacaagcagg
361 ctcctgggaa cctggcggca ggtgtgtaga accaaattgg ata
```

C

```
  1 aattcttcct ttggttttgc tgctttgcct ggttctgttt tcctattacc cttgattctc
 61 tgccttctag gggaaaagct caaattgtac ctaggactgc gttgaaatct gaggttgaaa
121 cttggtcctt gttgaaccat ttaggtggga aacacaaacc tggtctcctt tcccctctct
181 taaaggttct gtatgatggg cacgggaaac cccccaggct gcacccaaat gacaaacttt
241 tgccctacat aaaaaggctc tataaggcat acgctaccaa ggaggggacc cctaaatcca
301 acaaacccca cctctacaac actgttcggc tcttcacccc ctgtgctcag cacaagcagg
361 ctcctgggaa cctggcggca ggtgtgtaga accaaattgg a
```

D

```
  1 aattcttcct ttggttttgc tgctttgcct ggttctgttt tcctattagc cttgattctc
 61 tgccttctag gggagaagct caaattgtac ctaggactgc gttggaatct gaggctgaaa
121 cttggtcctt gttgaaccat ttaggtggga aacacaaacc tggtctcctt tcccctctct
181 taaaggttct gtatgatggg cacgggaaac cccccaggct gcacccaaat gacaaagctt
241 tgccctacat gaaaaggctc tataaggcat accctaccaa ggagggaacc cctaaatcca
301 acaaacccca cctctacaac actgttcggc tcttcacccc ctgtgctcac cacaagcagg
361 ctcctgggga cctggcggca ggtgtgtaga agcaaattgg a
```

E

```
  1 aattcttcct ttggttttgc tgctttgcct ggttctgttt tcctattacc cttgattctc
 61 tgccttctag gggaaaagct caaattgtac ctaggactgc gttggaatct gaggctgaaa
121 cttggtcctt gctgaaccat ttaggtggga aacacaaacc tggtctcctt tcccctctct
181 taaaggttct gtatgatggg cacgggaaac cccccaggct gcacccaaat gacaaagctt
241 tgccctacat gaaaaggctc tataaggcat acgctaccag ggaggggacc cctaaatcca
301 acaaacccca cctctacaac actgttcggc tcttcacccc ctgtgctcag cacaagcagg
361 ctcctgggga cctggcggca ggtgtgtaga agcaaattgg ata
```

F

```
  1 aattcttcct ttggttttgc tgctttgcct ggctctgttt tcctattacc cttgattctc
 61 tgccttctag gggaaaagct caaattgtac ctaggactgc gttgaaatct gaggctgaaa
121 cttggtcctt gctgaaccat ttaggtggaa aacacaaacc tggtctcctt tcccctctct
181 taaaggttct gtatgatggg cacgggaaac cccccaggct gcacccaaat gacaaagctt
241 tgccctacat gaaaaggctc tataaggcat acgctaccaa ggaggggacc cctaaatcca
301 acaaacccca cctctacaac actgttcggc tcttcacccc ctgtgctcac cacaaccagg
361 ctcctgggga cctggcggca ggtgtgtaga accaaattgg a
```

Fig.S3 (A) - A 462 bp sequence of GDF-9 gene of Rahmani breed *(NCBI accession no. KT357481.1)*, (B) - A 462 bp sequence of GDF-9 gene of Barki breed *(NCBI accession no. KT357482.1)*, (C) - A 462 bp sequence of GDF-9 gene of Rahmani X Barki cross breed *(NCBI accession no. KT357484.1)*, (D) - A 462 bp sequence of GDF-9 gene of Awassi breed *(NCBI accession no. KT357483.1)*, (E) - A 462 bp sequence of GDF-9 gene of Awassi X Suffolk cross breed *(NCBI accession no. KT357485.1)*, (F) - A 462 bp sequence of GDF-9 gene of Ossimi breed *(NCBI accession no. KT357486.1)*.
