# Supplementary material for: BMPR-1B, BMP-15 and GDF-9 genes structure and their relationship with litter size in six sheep breeds reared in Egypt
Source: BMC Res Notes. 2020 Apr 10;13:215. doi: 10.1186/s13104-020-05047-9 (PMC7160958; doi:10.1186/s13104-020-05047-9)
Supplement: Supplementary file 2 — Additional file 2: Table S1 The listing primer and sequence (5’→3’) of BMPR-1B, BMP-15, and GDF-9 genes. Table S2 Cycles conditions of PCR. Table S3 Nucleotide sequence distances: percent similarity (above diagonal), percent distance (below diagonal); of GDF-9 gene of tested sheep breeds. Table S4 The changed amino acids in tested sheep breeds. Table S5 Genotyping and allele frequencies analysis of the studied sheep breeds for BMPR-1B, and BMP-15 genes. [file 13104_2020_5047_MOESM2_ESM.pdf]

Table S1 The listing primer and sequence (5'→3') of BMPR-1B, BMP-15, and GDF-9 genes.

**Locus**	**Method**	**Primer sequence (5'→3')**	**Reference**
BMPR.1B (Fec-B)	RFLP	F:5′-CCAGAGGACAATAGCAAAGCAAA-3′<br>R:5′-CAAGATGTTTTCATGCCTCATCAACAGGTC-3′	Wilson *et al.*,2001
BMP-15 ($FecX^{G}$)	RFLP	F:5′CACTGTCTTCTTGTTACTGTATTTCAATGAGAC-3′<br>R:5′-GATGCAATACTGCCTGCTTG-3′	Hanrahn *et al.*, 2004
GDF-9 ($FecG^{H}$)	SSCP	F:5′- GAAGACTGGTATGGGGAAATG-3′<br>R:5′- CCAATCTGCTCCTACACACCT-3′	

Table S2 Cycles conditions of PCR

Locus	Denaturation		Annealing		extension		Final extension		Number of cycles
BMPR-1B (Fec-B)	°C	Sec	°C	Sec	°C	Sec	°C	Sec	**N**
	94	300	60	30	72	30	72	300	**30**
BMP-15 ($FecX^G$)	°C	Sec	°C	Sec	°C	Sec	°C	Sec	**N**
	94	300	62	40	72	45	72	300	**35**
GDF-9 ($FecG^H$)	°C	Sec	°C	Sec	°C	Sec	°C	Sec	**N**
	94	300	58	40	72	60	72	600	**35**

Table S3 Nucleotide sequence distances: percent similarity (above diagonal), percent distance (below diagonal); of GDF-9 gene of tested sheep breeds

Breeds	Rahmani	Barki	RXB*	Awassi	SXA**	Ossimi
Rahmani	-	0.98	0.97	0.97	0.97	0.98
Barki	0.0205	-	0.98	0.98	0.98	0.98
RXB*	0.0283	0.0179	-	0.97	0.98	0.97
Awassi	0.0283	0.0179	0.0309	-	0.98	0.97
SXA**	0.0257	0.0205	0.0230	0.0179	-	0.98
Ossimi	0.0179	0.0231	0.0257	0.0257	0.0179	-

*RXB = Rahmani **X** Barki cross
SXA = Awassi **X Suffolk cross

Table S4 The changed amino acids in tested sheep breeds

Breed/ amino acids	1	2	3	4
**1- Rahmani**	**(L)** Lysine	**(V)** Valine	**(E)** Glutamic	**(N)** Asparagine
**Other breeds**	**(R)** Arginine	**(G)** Glycine	**(G)** Glycine	**(K)** Lysine
**2- Barki**	**(F)** Phenylalanine	------	------	------
**Other breeds**	**(I)** Isoleucine	------	------	------
**3- RXB***	**(N)** Asparagine	**(F)** Phenylalanine	**(S)** Serine	------
**Other breeds**	**(K)** Lysine	**(L)** Leucine	**(T)** Threonine	------
**4- Awassi**	**(A)** Alanine	**(E)** Glutamic	**(S)** Serine	------
**Other breeds**	**(P)** Proline	**(G)** Glycine	**(T)** Threonine	------
**5- SXA****	**(G)** Glycine	**(S)** Serine	**(S)** Serine	------
**Other breeds**	**(R)** Arginine	**(T)** Threonine	**(T)** Threonine	------
**6- Ossimi**	**(T)** Threonine	------	------	------
**Other breeds**	**(S)** Serine	------	------	------

*RXB = Rahmani X Barki cross
**SXA = Awassi X Suffolk cross

Table S5 Genotyping and allele frequencies analysis of the studied sheep breeds for BMPR-1B, and BMP-15 genes

Breed	No. of samples	Genotype frequency					
		BMPR-1B (Fec-B)			BMP-15 (FecX ^{G})		
Method		PCR-RFLP			PCR-SSCP		
Allele		(BB)[3]	(B+)[4]	(++)[5]	(GG)[6]	(G+)[7]	(++)[8]
**Rahmany**	20	0	0	1	1	0	0
**Barki**	20	0	0	1	0	0	1
**RXB**[1]	69	0	0	1	0.58	0.29	0.13
**Awassi**	10	0	0	1	0	0	1
**AXS**[2]	8	0	0	1	0	0	1
**Ossimi**	5	0	0	1	0	0	1

[1]**RXB**; Rahmani X Barki cross. [2]**SXA**; Awassi X Suffolk cross. BMPR-1B gene had three different Booroola genotypes ([3]**BB**, [4]**B+** and [5]**++**) for (Fec-B) mutation, in the current study there is only one genotype "the wild-type (++)" with 190 bp band. BMP-15 gene had three different genotypes ([6]**GG**, [7]**G+** and [8]**++)** for (FecX^{G}) mutation, in the current study there are the three genotypes in Rahmani X Barki cross, and only one genotype in Rahmani breed (GG).
